# Using *Caenorhabditis elegans* to Uncover Conserved Functions of Omega-3 and Omega-6 Fatty Acids

**DOI:** 10.3390/jcm5020019

**Published:** 2016-02-02

**Authors:** Jennifer L. Watts

**Affiliations:** School of Molecular Biosciences and Center for Reproductive Biology, College of Veterinary Medicine, Washington State University, Pullman, WA 99164, USA; jwatts@vetmed.wsu.edu; Tel.: +1-509-335-8554; Fax: +1-509-335-4159

**Keywords:** *C. elegans*, polyunsaturated fatty acids, fat-1, omega-3 desaturase

## Abstract

The nematode *Caenorhabditis elegans* is a powerful model organism to study functions of polyunsaturated fatty acids. The ability to alter fatty acid composition with genetic manipulation and dietary supplementation permits the dissection of the roles of omega-3 and omega-6 fatty acids in many biological process including reproduction, aging and neurobiology. Studies in *C. elegans* to date have mostly identified overlapping functions of 20-carbon omega-6 and omega-3 fatty acids in reproduction and in neurons, however, specific roles for either omega-3 or omega-6 fatty acids are beginning to emerge. Recent findings with importance to human health include the identification of a conserved Cox-independent prostaglandin synthesis pathway, critical functions for cytochrome P450 derivatives of polyunsaturated fatty acids, the requirements for omega-6 and omega-3 fatty acids in sensory neurons, and the importance of fatty acid desaturation for long lifespan. Furthermore, the ability of *C. elegans* to interconvert omega-6 to omega-3 fatty acids using the FAT-1 omega-3 desaturase has been exploited in mammalian studies and biotechnology approaches to generate mammals capable of exogenous generation of omega-3 fatty acids.

## 1. Introduction

The adage “you are what you eat” is especially true regarding fat. Specific types of fat in the human diet have important biological consequences on health and wellness. Long chain polyunsaturated fatty acids (PUFAs) are dietary requirements for humans and other mammals. Essential fatty acids can be classified as omega-6, or omega-3, depending on the position of the terminal double bond relative to the methyl end of the fatty acids. For example, the omega-6 fatty acid linoleic acid (LA, 18:2) is an 18-carbon fatty acid with double bonds at carbons 9 and 12, while the omega-3 fatty acid alpha-linolenic acid (ALA, 18:3) is an 18-carbon fatty acid with double bonds at carbons 9, 12, and 15. The essential fatty acids can be elongated and further desaturated to generate a range of 20- and 22-carbon omega-6 and omega-3 fatty acids [1]. Omega-6 fatty acids are prevalent in vegetable oils, especially corn, safflower, and soybean oil, while omega-3 fatty acids are found in fishes such as salmon and tuna. Because humans cannot interconvert omega-6 and omega-3 fatty acids, the ratio of omega-6 to omega-3 fatty acids is determined by dietary intake [2]. This is important because, in spite of their structural similarity, the biological functions of omega-6 and omega-3 fatty acids can be quite divergent [3].

Long chain fatty acids from the omega-3 and omega-6 families play crucial roles in membrane structure and function [4]. For example, the *cis* double bonds influence lipid packing, membrane fluidity [5], and membrane protein activity [6]. Omega-3 and omega-6 fatty acids are precursors for potent signaling molecules, and signals produced from omega-3 *versus* omega-6 PUFAs can sometimes have opposing effects [3]. Upon stimulation, long chain omega-6 and omega-3 fatty acids are cleaved from membrane lipids by phospholipases and oxygenated by cyclooxygenase, lipoxygenase, or cytochrome P450 enzymes to form a wide range of prostaglandins, leukotrienes, lipoxins, as well as hydroxy-, epoxy-, and hydroperoxy-derivatives [7,8]. These molecules, collectively termed “eicosanoids” act as powerful short range hormones affecting inflammation, immune responses, and reproductive processes [9]. PUFAs are important components of endocannabinoids, which are ethanolamide derivatives of phospholipids that bind to endocannabinoid receptors to regulate memory, appetite, mood, and pain sensation [10,11]. Finally, both omega-3 and omega-6 PUFAs and their eicosanoid derivatives are ligands for transcription factors, and therefore they influence gene expression. In addition to known receptors for eicosanoid and ethanolamide derivatives, an omega-3 fatty acid receptor, GPR120, has recently been identified [12,13]. Substantial evidence exists for opposing functions of omega-3 and omega-6 fatty acids in the regulation of inflammation, primarily that eicosanoids derived from most omega-6 fatty acids have pro-inflammatory effects, while those derived from omega-3 fatty acids do not (reviewed in [3]). However, opposing functions for omega-3 and omega-6 fatty acids in non-inflammatory processes are not well defined.

## 2. Why Study Fatty Acid Functions Using *C. elegans*?

**Figure 1:**
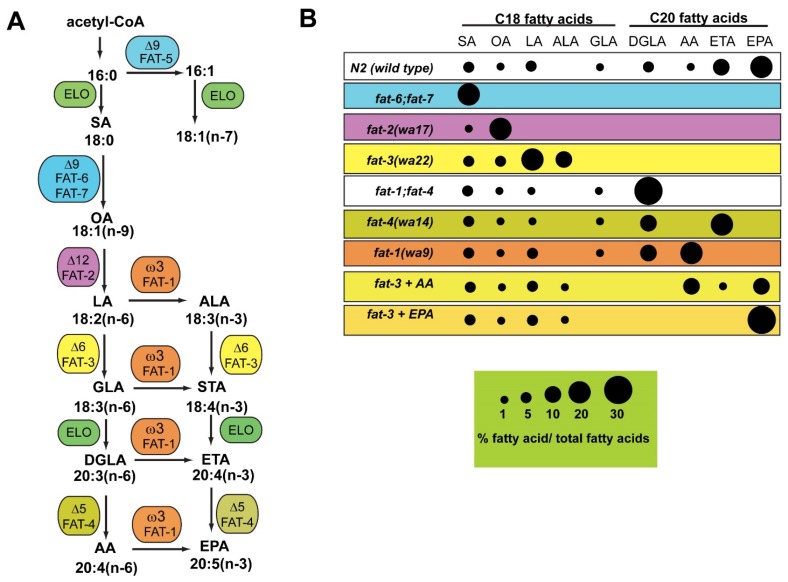
(**A**) Biosynthesis of omega-6 and omega-3 fatty acids in *C. elegans*. Unlike most other animals, *C. elegans* possesses ∆12 desaturase and ω3 desaturase enzymes, and therefore does not require essential dietary fatty acids to synthesize a range of omega-6 and omega-3 fatty acids; (**B**) Mutant strains lacking functional desaturase enzymes are depleted in specific polyunsaturated fatty acids. The area of each circle represents the ratio of specific fatty acids relative to total fatty acid content. Data shown are representative examples of multiple GC/MS measurements. Abbreviations: ELO, elongase; SA, stearic acid; OA, oleic acid; LA, linoleic acid; ALA, alpha-linolenic acid; GLA, gamma-linolenic acid; STA, stearidonic acid; DGLA, dihommo gamma-linolenic acid; ETA, eicosatrienoic acid; AA, arachidonic acid; EPA, eicosapentaenoic acid. Figure is modified from [23].

An attractive animal model for studies of fatty acid function is the roundworm *C. elegans*. This popular model organism is easy and inexpensive to maintain in the lab, and its well-understood developmental programs, simple anatomy, short lifespan, well-annotated genome, and ease of genetic analysis allow for studies of diverse biological processes, including those related to human nutrition and disease [14]. In the lab, the nematodes grow on petri plates on lawns of *Escherichia coli* bacteria, which provide dietary nutrients, including proteins, carbohydrates, and saturated and mono-unsaturated fatty acids derived from digestion of bacterial membranes [15]. However, *C. elegans* are capable of synthesizing all necessary fatty acids *de novo*, and the core enzymes of fatty acid biosynthesis are conserved, including acetyl CoA carboxylase, fatty acid synthase, and a range of fatty acid desaturase and elongase activities, enabling *C. elegans* to synthesize long chain PUFAs including arachidonic acid (AA, 20:4) and eicosapentaenoic acid (EPA, 20:5) [16] (Figure 1A). Unlike most other animal species, the *C. elegans* genome encodes an omega-3 desaturase enzyme that can convert 18-carbon and 20-carbon omega-6 fatty acids into omega-3 fatty acids [17], along with a ∆12 desaturase, which catalyzes the formation of LA from oleic acid (OA, 18:1) [18]. Thus, *C. elegans* does not have any dietary fatty acid requirements. Like most other animals, *C. elegans* also possesses ∆6 and ∆5 desaturase enzymes, which act, in conjunction with fatty acid elongases, on similar substrates used by mammals and other animals to form 20-carbon PUFAs [19]. However, *C. elegans* lacks the specific elongase activity to produce 22-carbon PUFAs. Strains containing mutations in genes of the fatty acid desaturation pathway facilitate functional studies of PUFAs, because fatty acid composition can be manipulated both genetically and through the diet [20,21,22].

## 3. *C. elegans* Mutants Lacking Fatty Acid Desaturase Activity Are Useful for Studies of Conserved Functions of Omega-6 and Omega-3 Fatty Acids

The *C. elegans* genome contains single genes encoding the ∆12, ∆5, ∆6, and omega-3 desaturase enzymes necessary for the biosynthesis of a range of omega-6 and omega-3 PUFAs. There are three genes encoding the ∆9 desaturases, which convert saturated fatty acids into monounsaturated fatty acids (MUFAs). One ∆9 desaturase, FAT-5, uses palmitic acid (16:0) as a substrate to produce palmitoleic acid (16:1), while the other two ∆9 desaturases, FAT-6 and FAT-7 use both 16:0 and stearic acid (18:0) as substrates to produce (16:1) and oleic acid (18:1) [24]. Mutant strains lacking functional desaturase enzymes have been isolated and used for many studies of functions of specific fatty acids in reproduction, longevity and neurobiology. Because roundworms do not have blood vessels, nor do they express inflammatory markers such as TNFα and NFκβ, the roles of omega-6 and omega-3 PUFAs can be studied independently of their inflammatory functions. As might be expected, strains carrying mutations in the desaturase genes acting early in the pathway, such as ∆9 desaturases (*fat-6;fat-7* double mutants), ∆12 desaturase (*fat-2* mutants), and ∆6 desaturase (*fat-3* mutants) have more severe lipid composition changes, as well as more severe phenotypic consequences, such as growth, movement, and reproductive defects, than strains carrying mutations in desaturases acting later in the pathway, such as the ∆5 desaturase [20,21,25] (Figure 1B). *C. elegans* has been a powerful model for unraveling mechanisms of development, neurobiology, and longevity, and the studies reviewed below demonstrate how fatty acid function is involved in these fundamental biological processes.

## 4. Functions of Omega-6 and Omega-3 Fatty Acids in *C. elegans* Reproduction: Sperm Guidance and Germ Cell Maintenance

PUFAs are required for efficient reproduction in many animal species, although specific mechanisms in invertebrates are just starting to be elucidated [26]. Initial characterization of *fat-2*, *fat-3* and *fat-6;fat-7* mutants lacking 20-carbon PUFAs revealed slow growth and greatly reduced reproductive capacity in the mutant strains [20,21,25]. It is likely that PUFAs are required for multiple processes to ensure optimum reproductive output. One process characterized by the Miller lab involves signaling molecules derived from PUFAs that are required in the female germ line for sperm guidance toward oocytes [27]. When wild-type males were mated to *fat-2* mutants, which are unable to synthesize PUFAs, the sperm failed to migrate toward the spermatheca, the region of the uterus where fertilization occurs. However, when *fat-2* mutants were provided dietary 20-carbon PUFAs, either omega-3 or omega-6 species, sperm migration greatly improved [27]. It is likely that the signaling is mediated by prostaglandin derivatives of omega-3 and omega-6 PUFAs, because directly injecting nanomolar concentrations of human F-series prostaglandins promoted sperm movement [28]. A range of F-series prostaglandins are synthesized in the *C. elegans* germ line, including those derived from both omega-3 and omega-6 PUFAs [29]. Interestingly, *C. elegans* does not encode a clear homolog of the mammalian cyclooxygenase enzyme that is the rate-limiting step of prostaglandin synthesis. Instead, metabolite analysis revealed an alternative prostaglandin synthesis pathway that does not involve prostaglandin-D or -E intermediates [29,30]. The Miller lab took full advantage of whole-organism genetic approaches to discover that the synthesis of F-series prostaglandins is regulated by both insulin signaling in the intestine and TGF-β signaling in sensory neurons in order to finely control reproductive output [28,31]. Both of these signaling pathways respond to food availability, thus, during food scarcity, prostaglandin biosynthesis is reduced, leading to reduced efficiency of sperm locating the fertilization site, ultimately resulting in a lower fertilization rate. Interestingly, the alternative Cox-independent prostaglandin pathway may be conserved in mammals, because F-class prostaglandins have been detected in Cox double knockout mice [31].

While 20-carbon omega-6 and omega-3 PUFAs are redundant in their ability to promote proper sperm migration to oocytes, a role for divergent activities of omega-6 and omega-3 PUFAs in reproduction is suggested by dietary studies. Watts and Browse discovered that dietary supplementation of the omega-6 PUFA dihomogamma linolenic acid (DGLA, 20:3*n*-6) resulted in sterility due to the destruction of germ cells [32]. Supplementation of arachidonic acid (AA, 20:4*n*-6) also led to germ cell death, although at a much higher concentration than DGLA. On the contrary, supplementation with omega-3 fatty acids, such as eicosapentaenoic acid (EPA, 20:5*n*-3) had no adverse effects on the nematodes [32]. Genetic analyses revealed a large number of gene mutations that altered the sensitivity to DGLA. For example, the *fat-1* mutant strain, which cannot convert omega-6 fatty acids to omega-3 fatty acids, was more sensitive to dietary DGLA than wild type [32]. On the other hand, stress resistant strains, such as the insulin-like growth factor receptor *daf-2* mutants, did not become sterile when exposed to DGLA [33]. The *daf-2* mutants showed increased transcription of genes involved in detoxification and stress resistance, suggesting that the negative effects of DGLA may be due to a toxic product derived from DGLA [33,34]. A recent study from the Watts lab showed that the negative effects of DGLA in the *C. elegans* germ line are likely due to the production of specific epoxide derivatives of DGLA, the synthesis of which depend on the cytochrome P450 (CYP) enzyme CYP-33E2 [35]. Direct injection of specific epoxides derived from DGLA, but not those derived from EPA, triggered germ cell abnormalities resembling cell fusion or failed cytokinesis, which ultimately lead to apoptosis and germ cell death. Interestingly, even though knockdown of CYP-33E2 in *C. elegans* led to reduced germ cell death and higher reproductive outputs during DGLA feeding, the CYP-33E2 knock-down worms had a lower brood size when fed a normal diet [35]. This suggests that CYP-33E2 may be producing beneficial epoxides from other PUFAs, such as EPA, that could be required for optimal reproduction.

## 5. Functions of Omega-6 and Omega-3 Fatty Acids in Longevity: Critical Roles for ∆9 Desaturases

Because of its small size, short lifespan, and ease of genetic manipulation, *C. elegans* has been a premier organism for discoveries of genetic and physiological mechanisms regulating aging and longevity [36,37]. Recent lipidomics and genetic studies suggest roles for specific fatty acids in promoting longevity in *C. elegans* [38]. PUFAs contain more double bonds than saturated or MUFAs, therefore they are more likely to undergo peroxidation, which in turn leads to propagation of reactive oxygen species (ROS), which cause further damage to proteins and nucleic acids. In many organisms the degree of membrane unsaturation negatively correlates with lifespan [39]. This concept is supported by a *C. elegans* study showing a correlation between long-lived mutants in the insulin signaling pathway and changes in unsaturated fatty acid composition [40]. The mutants examined had increased (MUFAs) with decreased PUFAs. Additionally, high concentrations of dietary fish oil was shown to lead to higher levels of lipid peroxidation products and shorter lifespan [40,41]. In contrast, another study showed that supplementation of omega-6 PUFAs activates autophagy and promotes long lifespan [42]. Furthermore, several long-lived mutant strains express higher levels of ∆9 desaturases than wild type [34]. The ∆9 desaturases synthesize MUFAs in mammals, but are the first step in the synthesis of PUFAs in *C. elegans*. Thus, the roles of PUFAs in aging are still not clear.

Importantly, the oxidative damage theory of aging, which states that the accumulation of molecular damage due to ROS is a key contributor to aging, is currently undergoing a paradigm shift based on experiments in *C. elegans* [43]. Studies in which worms were grown under oxygen levels ranging from 2%–40% did not show significant changes in lifespan [44]. In addition, growth of *C. elegans* in the presence of antioxidants did not always increase lifespan [43], and specific mutations in antioxidant genes did not always lead to reduced lifespan [43,45,46]. Furthermore, treatment with low levels of chemicals that induce ROS actually produced increased resistance to oxidative stress and increased lifespan through the process of stress-induced hormesis [47,48]. Thus, while it is clear that high levels of ROS cause cellular damage, regulated ROS release and fluctuations in ROS are important for inducing both signaling and protection pathways [49,50,51,52]. This might explain why lower concentrations of dietary fish oil led to slightly longer lifespans [41]. It is also crucial to consider that membranes undergo constant remodeling and turnover. A recent study demonstrated that PUFAs in *C. elegans* membranes turn over very rapidly, such that the majority of PUFAs and other membrane lipids are replaced each day, suggesting that oxidized fatty acids are rapidly removed [53]. Knock-down of ∆9 desaturase activity reduces membrane turn-over, which might lead to increased lipid peroxidation in spite of lower PUFA production [53]. It appears that the beneficial functions of omega-6 and omage-3 PUFAs might outweigh the potential for oxidative damage conferred by the high degree of unsaturation.

In animals, dietary resources must be allocated between reproduction and somatic maintenance, thus reproduction and life span are metabolically linked. In *C. elegans*, removal of the germ line results in worms with increased fat stores and longer lifespan (reviewed in [54,55]). Several recent studies implicate fatty acid metabolism in long lifespan in germ-line ablated animals. Two nuclear hormone receptor homologs, NHR-80 and NHR-49, regulate the expression of ∆9 desaturases [56,57], and these two nuclear receptor proteins, as well as the FAT-6 and FAT-7 ∆9 desaturases are required for long lifespan in animals lacking a germ line [58,59]. Additionally, the lysosomal lipase LIPL-4 and intact autophagy pathways are required for extended longevity in germ-line less nematodes [60,61]. A recent metabolomic analysis revealed that a specific lipid species, oleoylethanolamide, accumulates in worms over-expressing the lipase LIPL-4. This ethanolamide derivative of oleic acid then directly binds to the lysosomal lipid chaperone LPB-8, which is then translocated to the nucleus. Oleoylethanolamide also binds to and activates the nuclear receptor NHR-80, activating transcription of ∆9 desaturases and promoting longevity [62]. Thus, lipid signaling from the lysosome to the nucleus has long lasting physiological effects, including lifespan extension.

In humans, diets high in sugars lead to excess lipid storage and ultimately cause adverse health effects, including obesity, diabetes, and cardiovascular coronary heart disease [63,64]. In *C. elegans*, dietary glucose causes shortened lifespan [65,66]. Two transcriptional regulators of ∆9 desaturases, MDT-15 and SBP-1 were recently shown to protect *C. elegans* from glucose-induced accelerated aging by preventing the accumulation of saturated fat [67]. Glucose feeding increases the saturated fatty acid composition of *C. elegans*, and MDT-15 and SBP-1 activate ∆9 and other fatty acid desaturases to prevent saturated fatty acid-induced lipotoxicity by converting saturated fatty acids into MUFAs and PUFAs [67,68,69,70].

A key transcription factor required for increased longevity in germ-line ablated worms is SKN-1/Nrf. This transcription factor is most studied for its roles in stress responses, especially to oxidative stress [71]. Interestingly, SKN-1 also regulates the transcription of lipid metabolism genes that are up-regulated in germ-line deficient animals, including fatty acid desaturases [71]. When SKN-1 is over-expressed, lipids are depleted from somatic, but not germ-line tissues [72]. This phenotype is similar to that seen in wild type worms upon nutrient depletion or exposure to pro-oxidants, and is also seen in *fat-6;fat-7* double mutants lacking 20-carbon PUFAs, as well as in *fat-1;fat-4* double mutants, which accumulate DGLA, but cannot synthesize EPA or AA. Supplementation with OA, AA, and EPA, but not other fatty acids, suppressed the somatic depletion of lipids, suggesting that specific fatty acid species may be involved in the allocation of germline *versus* somatic lipids, thereby influencing both reproduction and longevity [72].

## 6. Functions of Omega-6 and Omega-3 Fatty Acids in *C. elegans* Neurons: Synaptic Vesicle Formation, Signal Transduction in Sensory Neurons, and Complex Behavioral Responses to Alcohol and Oxygen

Other than adipose tissue, human brain tissue contains the highest proportion of lipids, with brain phospholipids containing high levels of both omega-6 and omega-3 PUFAs. Diets deficient in omega-3 and other PUFAs lead to defective neural function (reviewed in [73,74]). *C. elegans* has a simplified nervous system consisting of 302 neurons, and the network of neurons and their connections has been thoroughly mapped [75]. The *fat-3* mutant strain, which lacks 20-carbon omega-6 and omega-3 PUFAs, shows uncoordinated movement and defective egg laying behavior, phenotypes which are controlled by motor neurons and hermaphrodite-specific serotonergic vulva neurons [21,76]. Lesa *et al*. showed that *fat-3* mutants display defects in neurotransmitter release, and that synaptic vesicles were depleted in neuronal termini at the neuromuscular junction, indicating that 20-carbon PUFAs are required for synaptic vesicle formation and accumulation [76].

Sensory neurons are also affected in *fat-3* and *fat-1;fat-4* double mutants, which exhibit defects in olfactory chemotaxis behavior to volatile odorants sensed by AWC neurons, but less or no defective chemotaxis behavior to odorants sensed by AWA neurons [23]. This genetic evidence implicates PUFAs in specific neuronal signal transduction pathways. The AWA neurons possess TRPV type channels to respond to stimulatory signals, whereas the AWC neurons, which are not affected by PUFA deficiency, use cyclic nucleotide gated channels. Similarly, the ASH sensory neurons use TRPV channels to respond to noxious stimuli, and stimulate rapid escape behavior to heavy metals, high osmolarity, and nose touch. The *fat-3* and *fat-1;fat-4* double mutants showed behavioral defects upon exposure to these stimuli, and were rescued by dietary supplementation of both omega-6 and omega-3 fatty acids, indicating that PUFAs are also necessary for function in ASH neurons [23]. Direct calcium imaging of ASH neuronal response in *fat-3* mutants showed diminished calcium responses, while exogenous EPA elicited calcium responses and avoidance responses in *fat-3* mutants, bypassing the PUFA biosynthesis defects, and providing evidence for 20-carbon omega-6 and omega-3 PUFAs as regulators of *in vivo* TRPV channel activity [23].

Olfactory adaptation is the process where sensory neurons reduce their response to prolonged stimulation. Even though sensory response to volatile odorants sensed by AWC neurons was nearly normal in PUFA-deficient mutants, adaptation to volatile odorants mediated by AWC neurons was abnormal in *fat-3* and *fat-1;fat-4* mutant strains, and this defect was rescued by dietary PUFAs [77]. Similar to the findings described above for AWA neurons, this study linked the roles of PUFAs in olfactory adaptation in AWC neurons to TRPV channels, which in AWC neurons function downstream of the nuclear accumulation of the cGMP-dependent protein kinase EGL-4 [77].

In the research described above, omega-6 and omega-3 fatty acids play redundant roles in their ability to rescue the neuronal defects, indicating that both omega-6 and omega-3 fatty acids perform the required cellular functions. Several recent studies suggest specific roles for omega-6 and omega-3 fatty acids in neuronal processes. A *C. elegans* mutant in fatty acid amide hydrolase activity (*faah-1*) is defective in the regeneration of axons after laser surgery [78]. Fatty acid amide hydrolase breaks down endocannabinoids, such as arachidonyl ethanolamide (AEA). In *C. elegans*, AEA appears to inhibit axon regeneration via the Goα subunit GOA-1 [78]. Surprisingly, eicosapentaenoyl ethanolamide (EPEA), derived from EPA, shows less inhibitory activity, even though EPEA is much more abundant in *C. elegans* tissues than AEA [79]. On the other hand, omega-3 fatty acids are specifically required for the process of alcohol tolerance [80], a neuroadaptive process that compensates for the effects of alcohol in humans and in *C. elegans* [81]. This study showed that unlike wild type nematodes, *fat-3*, *fat-4*, and *fat-1* mutants did not recover movement after exposure to ethanol. EPA, but not AA supplementation was necessary and sufficient for the neuroplasticity required to compensate for the effects of alcohol intoxication in *C. elegans* [80]. Finally, AA in phospholipids, but not EPA, is required for neurons responding to light touch [82]. The Goodman lab found that *fat-3* and *fat-4* mutants, neither of which can synthesize AA, showed reduced response to touch. AA, but not EPA, rescued touch sensitivity in *fat-3* and *fat-4* mutants. Interestingly, eicosatetraynoic acid (EYTA), a non-metabolizable structural analog of AA, also rescued the touch response phenotype. The ability of EYTA to rescue touch response indicates that AA is not required to be oxidized into an eicosanoid for its activity. In addition to mutant and dietary supplementation analysis, the researchers used dynamic force spectroscopy to reveal that AA in phospholipids modulates biophysical properties of touch receptor neuron membranes to allow for optimal function [82]. Taken together, these recent studies reveal that omega-6 and omega-3 PUFAs can have distinct roles in neurological processes.

In addition to the alternative prostaglandin synthesis pathway described above, recent studies demonstrate that in *C. elegans*, omega-3 and omega-6 fatty acids are modified into eicosanoid-like molecules by the actions of CYP enzymes, and that these CYP-derived eicosanoids have important biological functions. Studies from Menzel’s group revealed that CYP-33E2, which is most closely related to human CYP2J2, prefers EPA over AA as a substrate, and produces epoxyeicosatetraenoic acid (17,18-EEQ) as its main product [83]. CYP-29A3 is most closely related to human CYP4, and it uses AA to produce the hydroxyl derivative 20-HETE [84]. These eicosanoids have opposing effects on pharyngeal pumping and food uptake, with 17,18-EEQ mimicking the effects of the neurohormone serotonin on fasted worms, where both the eicosanoid and serotonin stimulate pharyngeal pumping. In contrast, 20-HETE and octopamine reduced pharyngeal pumping and food intact in well-fed worms [85]. Furthermore, 17,18-EEQ synthesis is increased upon serotonin supplementation, while 20-HETE synthesis increases upon application of octopamine [85], implicating eicosanoids as mediators of neurohormones affecting food intake. Another CYP enzyme, CYP-13A12, acts on PUFAs to respond oxygen levels [86]. In humans, reoxygenzation after oxygen deprivation causes tissue damage due to inflammation [87]. The *C. elegans* study showed that CYP-13A12 responds to the oxygen-dependent enzyme EGL-9 and hypoxia-inducible factor (HIF-1) to facilitate a movement response to reoxygenation after oxygen deprivation [86]. CYP-13A12 generates epoxy and hydroxyl eicosanoids from AA and EPA, including 14,15-epoxyeicosatrienoic acid (14,15-EET) from AA and 17,18-EEQ from EPA [88]. This research implicates conserved roles for omega-6 and omega-3 PUFAs and eicosanoid formation in ischemia and reperfusion.

## 7. Using the *C. elegans Fat-1* Gene for Studies in Mammals: Endogenous Omega-3 Functions and Biotechnology Applications

The discovery of the first animal omega-3 desaturase, the *C. elegans* FAT-1 desaturase [17], enabled Kang to construct the *fat-1* transgenic mouse, which expresses the *C. elegans* omega-3 desaturase and permits the conversion endogenous omega-6 to omega-3 fatty acids in mammalian tissues [89]. Lipidomic analysis revealed hundreds of specific lipid species that are altered between wild type and the *fat-1* mouse, including EPA and DHA-containing phospholipids and cholesterol esters, as well as many species of EPA-derived epoxides and diols formed via CYP enzymes [90]. Studies using the *fat-1* mouse model now appear in numerous publications in which researchers examined the effects of endogenously synthesized, as opposed to dietary, omega-3 fatty acids on a range of processes in a mammalian model. This research provides evidence that increased production of omega-3 PUFAs, coupled with a reduction in omega-6 PUFAs, protects mammals from a range of diseases, including cancer, diabetes and inflammatory diseases (reviewed in [91,92,93,94,95]). Recent studies reveal new insights into molecular mechanisms in which omega-3 PUFAs protect against various disease outcomes as diverse as diabetic neuropathy [96], fatty liver disease [97], pancreatic beta cell death [98], and vascular inflammation [99]. Thus, the *fat-1* mouse model promises to be central to unraveling the mechanisms of omega-3 and omega-6 fatty acids in health and disease.

The success of expressing the *C. elegans fat-1* omega-3 desaturase gene in mice led the way for expression of *fat-1* in other mammals. Because modern diets are thought to be deficient in omega-3 fatty acids, increasing omega-3 fatty acids in human food is desirable [100]. Concerns regarding heavy metal contamination of marine fish [101], as well as depletion of ocean fish stocks due to overfishing, lead to a desire for alternative sources of omega-3 fatty acids in human diets. The creation of transgenic farm animals, such as pigs [102,103,104] and cattle [105,106], set the stage for using nematode *fat-1* genes to someday provide milk and meat with higher omega-3 content to human consumers. However, more studies are needed to ensure the safety of food produced from transgenic animals. In addition, altering the omega-3/omega-6 ratio in farm animals might have adverse effects on their reproduction, as lower brood size was observed in mice expressing *fat-1* in their mammary glands [107].

## 8. Conclusions and Future Studies

Discoveries made using model organisms have had significant impact on human medicine. For example, *C. elegans* research has been crucial for the elucidation of genetic pathways underlying programmed cell death, longevity, and signal transduction pathways that occur during development as well as during carcinogenesis (reviewed in [14]). This research has led to the discovery of drug targets and novel therapeutics in humans. Studies in *C. elegans* regarding non-inflammatory functions of PUFAs clearly demonstrate that 20-carbon PUFAs play key roles in reproduction and in the nervous system. While it appears that many functions of 20-carbon omega-6 and omega-3 fatty acids are redundant, examples of specific functions for DGLA, AA and EPA are beginning to emerge [35,42,80,82,85]. Genetic analysis and simple physiology render the *C. elegans* model especially useful for studies of PUFA functions in reproduction, development, and longevity, because vertebrate models are much more difficult, time consuming, and expensive to manipulate. Roles of PUFAs in longevity are just starting to be examined, and several studies suggest that ∆9 desaturase is a pro-longevity factor. Future studies are needed to determine if PUFA activity depends on being metabolized into eicosanoids or other signaling molecules, or whether their functions are derived from membrane biophysical properties or direct interactions with membrane proteins. Similarly, the identification of *C. elegans* receptors for PUFAs and eicosanoids, as well as the identification of specific signal transduction pathways that are affected by PUFA composition, will allow for more mechanistic studies. It is likely that mammalian studies using the *fat-1* transgenic mouse will continue to be fruitful because the transgene can be crossed into numerous genetic models of disease, thereby examining the effects of endogenous omega-3 fatty acid production on many different disease outcomes. In summary, *C. elegans* is a powerful model for the integration of dietary and genetic studies for PUFA function in reproduction, development, neuroscience and aging.
